# Incorporation of inosine into DNA by human polymerase eta (Polη): kinetics of nucleotide misincorporation and structural basis for the mutagenicity

**DOI:** 10.1042/BCJ20230159

**Published:** 2023-09-25

**Authors:** Qi Zhang, Natalia Tretyakova

**Affiliations:** Department of Medicinal Chemistry, University of Minnesota-Twin Cities, Minneapolis, U.S.A

**Keywords:** crystallography, DNA damage, DNA replication and recombination, DNA synthesis and repair, point mutations

## Abstract

Inosine, a purine nucleoside containing the hypoxanthine (HX) nucleobase, can form in DNA via hydrolytic deamination of adenine. Due to its structural similarity to guanine and the geometry of Watson–Crick base pairs, inosine can mispair with cytosine upon catalysis by DNA polymerases, leading to AT → GC mutations. Additionally, inosine plays an essential role in purine nucleotide biosynthesis, and inosine triphosphate is present in living cells. In a recent publication, Averill and Jung examined the possibility of polη catalyzed incorporation of deoxyinosine triphosphate (dITP) across dC and dT in a DNA template. They found that dITP can be incorporated across C or T, with the ratio of 13.7. X ray crystallography studies revealed that the mutagenic incorporation of dITP by human polη was affected by several factors including base pair geometry in the active site of the polymerase, tautomerization of nucleobases, and the interaction of the incoming dITP nucleotide with active site residues of polη. This study demonstrates that TLS incorporation of inosine monophosphate (IMP) into growing DNA chains contributes to its mutagenic potential in cells.

## Introduction

Inosine, a purine nucleoside containing hypoxanthine (HX) nucleobase, was first identified in 1965 as the deamination product of adenosine [1]. Inosine formation in RNA is a major biological mechanism underlying RNA editing [1]. Additionally, inosine has an important role in controlling primordial ribozyme activities in RNAs and improving the efficiency and accuracy of non-enzymatic RNA replication [2–4]. Inosine triphosphate (ITP) is found in many animal tissues and is a key intermediate in purine nucleotide biosynthesis and metabolism [1]. ITP is generated by pyrophosphorylation or stepwise phosphorylation of IMP, an essential metabolite of purine biosynthesis and a precursor of both AMP and GMP [1,5].

The deoxyribonucleotide dITP may be generated from dATP by non-enzymatic hydrolysis or by reduction in ITP [5]. However, the accumulation of inosine may cause human diseases and the accumulation of its triphosphate form (dITP) could lead to the misincorporation of inosine into DNA strands opposite C [1,6–9], causing AT → GC mutations.

Polymerase eta (Polη) belongs to the Y-family of DNA polymerases which can bypass DNA lesions in translesion synthesis (TLS). Following deubiquitination of the PCNA sliding clamp, normal DNA replication switches to a low-fidelity mode of DNA damage tolerance (DDT), which involves TLS polymerases such as polη [10]. Polη contains 713 amino acids with the catalytic domains including ‘finger', ‘palm', ‘PAD' (also named ‘little finger') and ‘thumb' [10–13]. Unlike replicative DNA polymerases which can be blocked in the presence of DNA lesions, polη has smaller ‘thumb' and ‘finger' domains, resulting in a large active site able to accommodate lesion-containing DNA substrates [10]. Lesion bypass by polη is mediated by several factors including interactions of the incoming nucleotide with Mg^2+^ or Mn^2+^ in the active site, syn-anti conformational equilibrium of the nucleotides, keto-enol tautomerization of the nucleobases, and interactions of the incoming deoxyribonucleotides with specific residues of polη during mutagenic bypass of DNA lesions [13]. Although polη and other TLS polymerases allow for bypass of DNA lesions during replication to avoid cell death, they may contribute to mutagenesis due to their ‘error-prone' nature and also increase the survival of cancer cells following treatment with common chemotherapeutic agents [10]. Therefore, the development of anticancer agents which can inhibit the TLS pathway has received additional attention in recent years [14–16].

The mutagenic potential of deoxyinosine present within DNA strands has been previously investigated, followed by crystallography studies of the polη tertiary complex with incoming nucleotides [17]. The HX base has been shown to form a Watson–Crick base pair with incoming dCTP in the active site of polη, allowing for exclusive incorporation of C into the DNA strand across inosine on the template. In contrast, the mutagenic and error-free incorporation of dITP into growing DNA chains had not been previously reported.

In a recent study, Averill and Jung investigated the incorporation of dITP into the growing DNA chain by human polη and studied its mutagenic potential [18]. These authors also elucidated the crystal structures of the polη tertiary complex containing incoming dITP to explain its error-prone incorporation by TLS polymerase. In their work, they first investigated the steady-state kinetics of the incorporation of dITP opposite unmodified C or T upon catalysis by human polη. They found that incorporation of dITP across unmodified C was less efficient than the incorporation of correct base (dGTP), and the incorporation of dITP across the unmodified T was even less efficient. However, the incorporation efficiency of dITP across dC was only 14-fold higher than across dT, revealing a potential novel mutagenic mechanism for inosine. This is in contrast with the exclusive incorporation of dC opposite HX on DNA template [17]. The observed differences in the mutagenic potential of inosine when it is present in the incoming nucleotide vs. in the DNA template was explained by crystal structure study of polη in complex with DNA template and incoming dITP.

## Mutagenic potential of the incorporation of dITP across dC or dT via steady-state kinetic study

Evaluating the catalytic efficiency of the DNA polymerases during TLS is critical for our understanding of mutagenic and error-free polymerase bypass in the presence of structurally modified DNA bases [17–22]. The ratio of *k*_cat_/K_M_ (catalytic efficiency) is a useful index for comparing the ability of DNA enzyme to incorporate specific nucleotides opposite native and modified DNA bases [23]. Previous steady-state kinetic study [17] reported that polη inserted dC opposite to HX with 37.4 × 10^−3^ s^−1^ µM^−1^ catalytic efficiency and dT with 0.54 × 10^−3^ s^−1^ µM^−1^ catalytic efficiency, in terms of *k*_cat_/K_M_ (Table 1). The relative efficiency in dC or dT incorporation opposite HX was about 69 : 1 (the ratio of *k*_cat_/K_M_ values) indicating nearly exclusive incorporation of dC across HX.

**Table 1:** Catalytic efficiency of nucleotide incorporation by human polη [18]

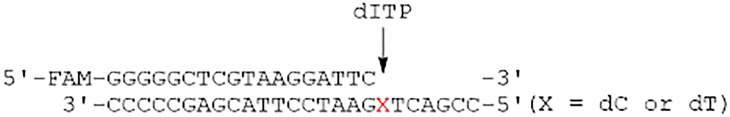			
Entry	Template:dNTP	*k*_cat_/K_M_ (10^−3^ s^−1^ µM^−1^)	Replication fidelity
1	dC:dITP	7.17	13.8
2	dT:dITP	0.52	1
3	dC:dGTP	18.5	185
4	dC:dATP	0.10	1
5	dT:dATP	19.9	180.9
6	dT:dGTP	0.11	1
7	HX:dCTP^1^	37.4	69.3
8	HX:dTTP^1^	0.54	1

1 Ref. [17].

In their elegant study, Averill and Jung investigated the incorporation of dITP (deoxynucleotide triphosphate form of inosine) across unmodified C or T by polη (Table 1). The catalytic efficiency for dITP incorporation opposite C was 7.17 × 10^−3^ s^−1^ µM^−1^ and opposite dT was 0.52 × 10^−3^ s^−1^ µM^−1^. In contrast with polη-mediated pyrimidine nucleotide incorporation across HX, the incorporation of dITP across dC was only around 14-fold more efficient than its incorporation across dT (7.17 × 10^−3^ s^−1^ µM^−1^: 0.52 × 10^−3^ s^−1^ µM^−1^). The incorporation of dC across HX and dITP across C had a lower fidelity than the incorporation of dGTP opposite C or dATP opposite T (Table 1). Moreover, although the incorporation of dITP across dT was less efficient than the incorporation across dC, it was more efficient than the incorporation of dT across HX (Table 1). The intriguing results reveal the dual mutagenicity potential of inosine depending on whether it is incorporated as dITP or is present in the DNA template. When dITP is incorporated across from C, it can lead to G to A transitions, while the incorporation of dITP across from T is not mutagenic. dITP addition opposite C is 14-fold more likely than its incorporation opposite T (Table 1). In contrast, HX present in DNA almost exclusively pairs with dCTP, leading to A to G transitions (Table 1) [17].

## Explanation of the mutagenicity of dITP incorporation by crystal structures of polη-nucleotide complex

To provide a structural explanation of the observed mutagenic and nonmutagenic incorporation of dITP into DNA substrates by polη during replication, Averill and Jung elucidated the crystal structures of polη-dC:dITP and polη-dT:dITP tertiary complexes. Both Watson-Crick base pair and wobble base pair were observed upon dITP incorporation across the templating C or T by using different tautomers of the bases. dC on the template can pair with the incoming dITP not only in Watson–Crick geometry but also via a wobble base pair, leading to less efficient incorporation by polη as compared with the incorporation of dGTP across dC (7.17 × 10^−3^ s^−1^ µM^−1^ vs. 18.5 × 10^−3^ s^−1^ µM^−1^). Although two possible base pair mechanisms were also observed with the incorporation of dITP across dT, only one catalytic metal was found in the active site, leading to weaker interactions with the incoming nucleotide and reduced incorporation efficiency as compared with dC:dITP incorporation (0.52 × 10^−3^ s^−1^ µM^−1^ vs. 7.17 × 10^−3^ s^−1^ µM^−1^). However, the dT:dITP incorporation was still more efficient than the incorrect dT:dGTP incorporation (0.52 × 10^−3^ s^−1^ µM^−1^ vs. 0.11 × 10^−3^ s^−1^ µM^−1^). On the other hand, the crystal structures of polη-base pair complex revealed differences in interactions of dITP with polη residues Gln38 and Ile48. The less stable interaction with Gln38 and further distance of base pair towards Ile48 leads to the dT:dITP incorporation being less efficient than dC:dITP. The overlapped structures of polη-dC:dITP, polη-dC:dGTP and polη-HX:dCTP indicate deviations in phosphodiester bond geometry, which causes less efficient incorporation of dITP across dC.

## Three factors contribute to the catalytic efficiency of polη for the mutagenic incorporation of dITP

Three main factors considered by Averill and Jung to influence the mutagenic potential of dITP incorporation by polη were polη: base pair geometry, tautomerization of thymine and cytosine bases, and the interactions of the incoming nucleotide with active site residues of polη. The mixed base pair of dC:dITP was less optimal than the canonical Watson–Crick base pair of dC:dGTP and HX:dCTP in the active site of polη, causing reduced catalytic efficiency of polη for dITP incorporation across dC. Incorporation of dITP across dT was more efficient than the misincorporation of dT:dGTP, since the latter has difficulty in the formation of stable hydrogen bonds between templating thymine and the incoming dGTP in the active site of polη. Second, the minor tautomer of dT forms a Watson–Crick-like base pair with dITP, leading to a more efficient incorporation of dITP opposite to dT as compared with dT: dGTP. The minor tautomer of cytosine on the template strand can form a wobble base pair with dITP, causing lower efficiency of dITP incorporation opposite C (as compared with dC:dGTP). Gln38 residue of polη facilitates the production of the mismatched base pair [17,24,25], affecting the base pair formation of templating dC or dT with dITP. Gln38 interacts with cytosine via hydrogen bonding in polη-dC:dITP complex and van der Waals interaction in polη-dT:dITP structure. All three factors contribute to the mutagenic incorporation of dITP by polη.

In conclusion, the recent study by Averill and Jung reports that mutagenic potential of inosine is mediated by the incorporation of dITP across dC or dT by TLS polη. The authors presented data for increased efficiency of the incorporation of dITP across dT as compared with the incorrect incorporation of dGTP across dT and elucidated the structural origins for incorporation of dITP across pyrimidine via the study of crystal structures of the polη tertiary complex. Their work provides evidence for the interesting dual mutation potential of inosine based on the use of dITP during oligonucleotide extension during DNA translesion synthesis. The study contributes to our understanding of the role of inosine in mutagenicity and encourages future investigations of inosine-mediated mutations *in vivo*.

## Open Access

Open access for this article was enabled by the participation of University of Minnesota in an all-inclusive *Read & Publish* agreement with Portland Press and the Biochemical Society under a transformative agreement with Individual.

## Abbreviations

HX: hypoxanthine
IMP: inosine monophosphate
ITP: inosine triphosphate
TLS: translesion synthesis
